# Comparative analysis of the gut microbiota composition among three captive hornbills

**DOI:** 10.3389/fmicb.2025.1642332

**Published:** 2025-08-07

**Authors:** Enmei Yang, Song Wang, Huajuan Feng, Fanglin Zheng, Yubao Duan, Shuang Yang

**Affiliations:** 1 The Key Laboratory of Forest Resources Conservation and Utilization in the Southwest Mountains of China Ministry of Education, Southwest Forestry University, Kunming, China; 2 Key Laboratory of National Forestry and Grassland Administration on Biodiversity Conservation in Southwest China, Southwest Forestry University, Kunming, China; 3 Key Laboratory for Forest Resources Conservation and Utilization in the Southwest Mountains of China, Ministry of Education, Southwest Forestry University, Kunming, China; 4 Key Laboratory of Forest Disaster Warning and Control in Yunnan Province, Southwest Forestry University, Kunming, China; 5 Key Laboratory for Conserving Wildlife with Small Populations in Yunnan, College of Forestry, Southwest Forestry University, Kunming, China; 6 Nanning Zoo, Nanning, China

**Keywords:** hornbills, gut microbiota, diversity, 16s rRNA sequencing technology, gut microbiota composition

## Abstract

This study investigated the gut microbiota of three captive hornbill species (*Anthracoceros albirostris, Buceros bicornis, Rhyticeros undulatus*) at the Nanning Zoo. Fecal samples were aseptically collected from 30 hornbills, and 16S rRNA high-throughput sequencing was employed to analyze the composition, diversity, and potential functions of the gut microbiota. The influence of host taxonomic status, sex, and captive environment on the gut microbiota was explored. The results revealed that the dominant phyla in the gut microbiota of all three hornbill species were Proteobacteria, Firmicutes, Actinobacteria, and Bacteroidetes. Although no significant differences were detected in the gut microbiota composition among different species and sexes, LEfSe analysis indicated significant enrichment of Erysipelotrichaceae and Lactobacillus_hayakitensis_DSM_18933_JCM_14209 in the Oriental Pied Hornbill group, as well as Clostridiales, Paenibacillaceae, and Paenibacillus_xylanilyticus in the Great Hornbill group. PICRUSt analysis indicated that the functional genes of the gut microbiota in all three hornbill species were primarily enriched in metabolic pathways, showing similar relative abundances. This study suggests that the gut microbiota characteristics of hornbills align with those of omnivorous birds. The homogenization of food resources in a captive environment may weaken the impact of host differences on the gut microbiota. The research findings provide a scientific basis for the health management of captive hornbills and the microecological assessment of wild population habitats.

## 1 Introduction

The intestines serve as the area where animals are most closely connected to their external environment. They play a crucial role in the digestion and absorption of nutrients, as well as in material exchange. Additionally, the intestines provide a suitable habitat for various microorganisms, including bacteria and fungi, making them a key site for interactions between animals and microbes (Kohl, 2012; Zommiti and Feuilloley, 2025). The gut microbiota is diverse and includes bacterial, fungal, and protozoan communities, with bacteria typically being the dominant group (Bodawatta et al., 2022). It is generally believed that gut microbes primarily originate from the mother, influenced by factors such as the birth process and breastfeeding (Dominguez-Bello et al., 2010), exposure to the surrounding environment after birth (Jones et al., 2018), and the host’s dietary intake (Li et al., 2016).

The composition and abundance of the host gut microbiota are jointly regulated by both intrinsic factors—such as the host’s physiological state (Ofek et al., 2022), age (Kubinyi et al., 2020), and sex (Li et al., 2021)—and external conditions, including environmental factors (Wang et al., 2023) and diet (Kudo et al., 2019). These symbiotic microorganisms participate in regulating key physiological functions such as nutrient metabolism, immune responses, neurobehavioral processes, and growth and development, thereby assisting the host in adapting to external pressures such as fluctuations in nutrient intake, environmental temperature changes, and pathogenic microbial invasions (Zommiti and Feuilloley, 2025). Due to their close association with host physiological functions, current research on gut microbes—particularly bacteria—primarily focuses on species systems closely related to human health and economically significant livestock, forming a relatively systematic scientific framework. For example, Ding et al. investigated the origins and establishment of avian embryonic gut microbiota using chickens as a model (Ding et al., 2017).

As research on gut microbiota deepens, the focus has gradually expanded from humans and model animals (such as mice and livestock) to wild mammals like primates (Zou et al., 2024; Greene et al., 2022). However, previous studies have predominantly concentrated on mammalian species, with limited research on non-mammalian taxa. Birds represent an exciting model system because their lineage encompasses over 10,000 species. The diversity among bird species—in migration behavior, flight capability, diet, mating systems, lifespan, and physiological traits—collectively shapes their gut microbiota. Due to their unique life history traits (e.g., migration, flight-related metabolic demands, dietary specialization, and reproductive strategies), birds provide a robust model for studying host-microbe interactions (Grond et al., 2018).

For instance, the impact of migration strategies (e.g., long-distance migrants in temperate zones versus resident tropical species) on microbial community structure appears relatively weak, whereas environmental fluctuations within climate zones (such as seasonal changes) may exert stronger effects on gut microbiota composition than geographic isolation across different climate zones (Schmiedová et al., 2023). Dietary differentiation (e.g., frugivory, insectivory, or omnivory) directly shapes the functional specialization of microbial communities (Wang et al., 2022). These differences govern interactions between birds and their gut microbiota, leading to alterations in microbial composition and structure.

Moreover, compared to mammals, birds typically have shorter intestines and lack a colon, resulting in significantly reduced gastrointestinal transit times. In terms of digestive physiology, birds lack teeth and cannot chew food; instead, they store and soften food in the crop. Their stomachs are divided into the proventriculus and gizzard: the proventriculus secretes digestive fluids, while the gizzard grinds food with ingested grit (Zheng, 2012). This unique digestive mechanism differs markedly from that of mammals. The diverse reproductive strategies of birds also offer new perspectives for studying vertical transmission of gut microbes, yet research on the gut microbiota of many endangered bird species remains limited.

**FIGURE 1:**
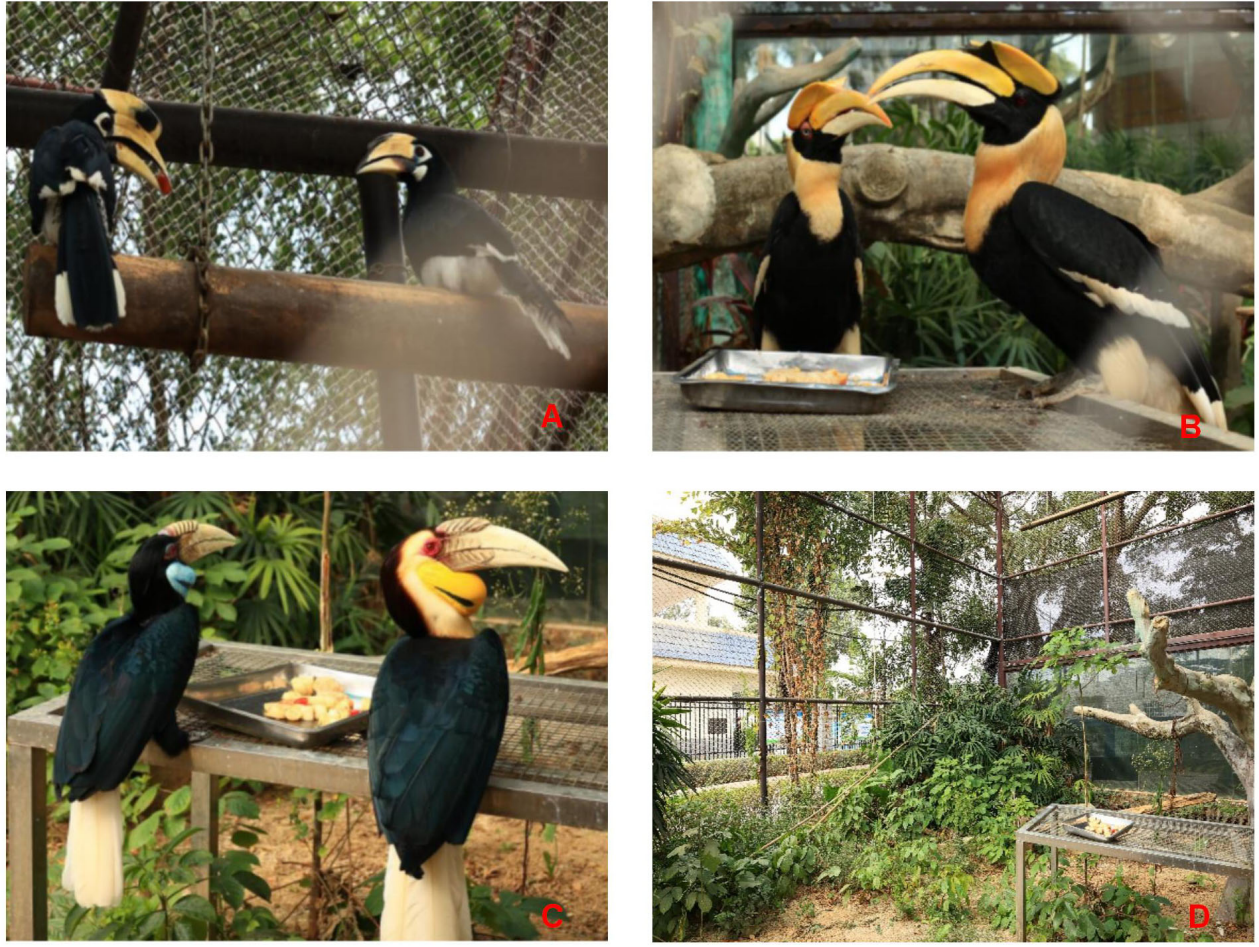
Oriental Pied Hornbill: male (left) and female (right) **(A)**; Great Hornbill: female (left) and male (right) **(B)**; Wreathed Hornbill: female (left) and male (right) **(C)**; Enclosure environments of the three hornbill species **(D).**

The Oriental Pied Hornbill (*Anthracoceros albirostris*), Great Hornbill (*Buceros bicornis*), and Wreathed Hornbill (*Rhyticeros undulatus*) are classified under the class Aves, order Bucerotiformes, and family Bucerotidae. All three species are designated as Class I National Key Protected Wild Animals^1^ (Figure 1). Their distribution exhibits significant regional specificity: the Oriental Pied Hornbill is primarily found in the karst seasonal rainforests and evergreen broad-leaved forests of Yingjiang and Mengla in Yunnan, and Fusui in Guangxi; the Great Hornbill mainly inhabits the mid-mountain humid evergreen broad-leaved forests of Yingjiang, Gengma, Cangyuan, Mengla, and Jinghong in southern Yunnan, extending to the subtropical monsoon forests of Motuo, Tibet; the Wreathed Hornbill is only observed in the Tongbiguan National Nature Reserve of Yingjiang, Yunnan, and in localized tropical rainforest habitats of Ruili (Zheng, 2023). All three species are highly dependent on intact tropical and subtropical forest ecosystems, utilizing tree cavities in tall trees for nesting and consuming fruits from plants like Ficus and insects as their primary food sources. Current research on hornbills primarily focuses on distribution (Trisurat et al., 2013), population size (Lubis et al., 2023), and breeding ecology (Gicaraya and Española, 2024). There is a significant lack of research on their gut microbiota, both in terms of microbial classification and functional aspects. More systematic studies are urgently needed. However, due to biological constraints such as habitat fragmentation, low population density, and secretive breeding behaviors in wild hornbills, systematic collection of field samples is extremely difficult. Although previous studies by Sun et al. have examined the gut microbiota of Great Hornbills and Wreathed Hornbills at Nanjing Hongshan Zoo, our current research investigates three hornbill species, including the Oriental Pied Hornbill. Additionally, the diets provided to hornbills differed significantly between the two zoos (Sun et al., 2019). Therefore, this study focused on captive individuals at Nanning Zoo, where fecal samples were aseptically collected under standardized husbandry conditions. Using high-throughput 16S rRNA gene sequencing, we analyzed gut microbial composition to compare diversity, taxonomic profiles, and potential functions across the three species. This approach elucidates the influence of host taxonomy, sex, and captive environment on microbiota assembly, providing a scientific basis for the health management of captive rhinoceros hornbills.

## 2 Materials and methods

### 2.1 Study site

The sampling for this study took place at Nanning Zoo, located in Xixiangtang District, Nanning City, Guangxi Zhuang Autonomous Region. This study selected three hornbill species within the park that can reliably provide samples: oriental Pied Hornbill, Great Hornbill, and Wreathed Hornbill. The zoo population included 14 Oriental Pied Hornbills, 18 Great Hornbills, and 9 Wreathed Hornbills. A total of 30 fecal samples were collected from these birds (Supplementary Table 1).

### 2.2 Fecal sample collection

The three hornbill species were housed in separate enclosures (Figure 1D) and were fed identical diets daily (consistent with the sampling day). To simulate the omnivorous diet of wild hornbills and facilitate dietary management and health assurance in captivity, their food includes rice balls (cooked rice and minced pork prepared separately), bananas, tomatoes, grapes, apples, and mealworms. However, these foods are not part of the natural diet of wild hornbill populations. Therefore, due to the captive environment and homogenization of diet, the research findings may not be generalizable to wild populations. Feeding occurred at approximately 9:00-10:00 a.m., 11:30 a.m.-12:00 p.m., and 4:00-5:00 p.m. each day. Fresh defecation events were monitored after feeding. Before sampling, sterile plastic sheets were placed beneath the perches. Immediately after defecation, the location was marked, and samples were collected to prevent contamination or degradation. During collection, disposable gloves were worn to retrieve fecal matter manually. Surface layers in contact with the ground or potentially contaminated by leaves or soil were carefully removed. Samples were promptly transferred into 5 mL sterile centrifuge tubes, labeled, and stored at −80°C freezers on-site. Subsequently, samples were transported to the laboratory on dry ice.

### 2.3 DNA extraction, amplification, and sequencing

Fecal sample DNA extraction was performed using the TGuide S96 Magnetic Bead-based Fecal Genomic DNA Extraction Kit (Model DP812) from TianGen Biotech. The detailed extraction procedure was carried out in accordance with the manufacturer’s instructions. Briefly, 0.25–0.5 g of fecal sample (or200 μL of liquid sample) was homogenized with 500 μL of lysis buffer SA, 100 μL of buffer SC containing proteinase K, and 0.25 g of grinding beads, followed by incubation at 70°C for 15 min for complete lysis. Subsequent steps included centrifugation, addition of nucleic acid binding buffer SH and GFA, and magnetic bead-based purification to obtain high-quality genomic DNA.

The V3-V4 hypervariable region of the bacterial 16S rRNA gene was amplified via PCR using purified DNA as the template, with universal primers 338F (5′-ACTCCTACGGGAGGCAGCA-3′) and 806R (5′-GGACTACHVGGGTWTCTAAT-3′). The PCR products were purified using VAHTSTM DNA Clean Beads and subjected to paired-end sequencing on an Illumina NovaSeq 6000 platform (Biomarker Technologies, China).

### 2.4 Data analysis

All fecal samples were divided into three groups; the first group compared the three species: different species: oriental Pied Hornbill (O), Great Hornbill (G), and Wreathed Hornbill (W), the remaining two groups compared the different sexes of the Oriental Pied Hornbill and Great Hornbill with females denoted as F and males denoted as M. (Wreathed Hornbill males were excluded due to a sample size of only two individuals). Bioinformatic analysis of the sequencing data included the following key steps: (1) Use Trimmomatic v0.33 to perform quality filtering on the raw sequencing data, setting the Phred quality score threshold to 20, followed by primer sequence identification and removal with Cutadapt 1.9.1. Denoising was carried out using the dada2 method in QIIME2 2020.6, along with paired-end sequence merging and chimera removal to obtain the final effective data. (2) Taxonomic annotation of feature sequences was done using a naive Bayesian classifier together with the SILVA reference database. (3) Alpha diversity indices (Chao1, Shannon, Simpson, and ACE indices) were computed using QIIME2 software to analyze the gut microbiota richness and diversity across different species. For grouped samples (with a minimum of three samples per group), the significance of differences was assessed using the Wilcoxon Rank Sum Test, with a significance level set at 0.05. Beta diversity analysis was conducted using QIIME based on weighted unifrac, with sample similarity visualized using NMDS. ANOSIM similarity analysis was performed using the “Vegan” package in R 4.0.2, with a significance level set at 0.05. (4) LEfSe (Line Discriminant Analysis Effect Size) analysis is a statistical method that combines non-parametric tests such as Kruskal-Wallis and Wilcoxon rank-sum with linear discriminant analysis (LDA) effect size measurement. It is used to identify biomarkers with statistically significant differences across different groups. LEfSe analysis was utilized to identify significantly different biomarkers between groups through linear discriminant analysis (LDA) and effect size estimation. (5) PICRUSt2 (Phylogenetic Investigation of Communities by Reconstruction of Unobserved States) is a bioinformatics tool that predicts functional potential based on the relative abundance of marker gene sequences in microbial community samples. The PICRUSt software was used to infer the functional gene composition of the samples based on the taxonomic composition information obtained from 16S rRNA sequencing data, enabling the analysis of functional differences between different groups.

## 3 Results and analysis

### 3.1 Sequencing results statistics

**FIGURE 2:**
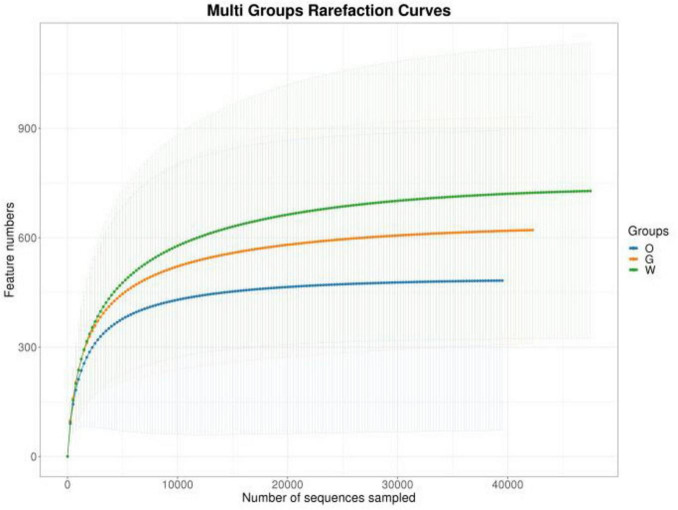
Sparsity curves for the three hornbill fecal samples.

**FIGURE 3:**
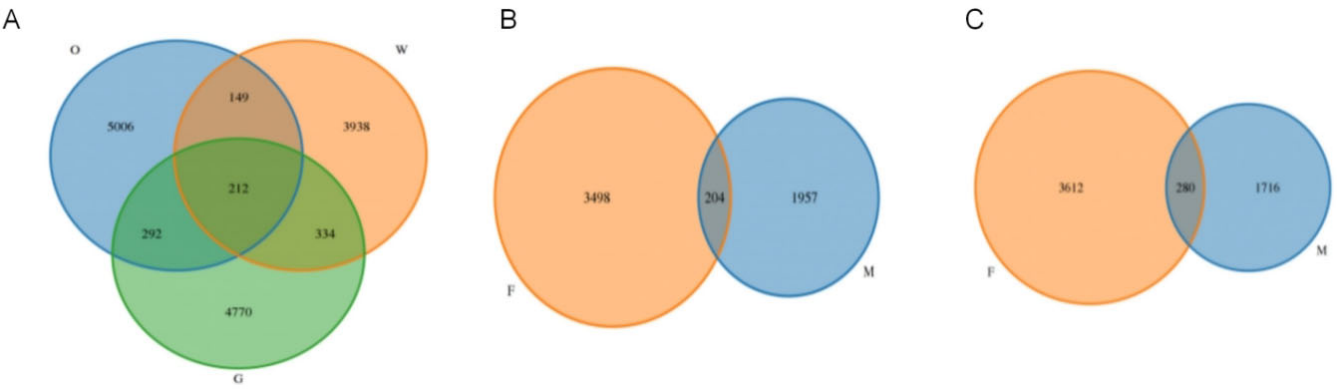
Venn diagrams illustrating the distribution of operational taxonomic units (OTUs) in the gut microbiota of three hornbill species (O: Oriental Pied Hornbills, G: Great Hornbills, W: Wreathed Hornbill) **(A)**; OTU distribution in the gut microbiota of male and female Oriental Pied Hornbills (F: female, M: male) **(B)**; OTU distribution in the gut microbiota of male and female Great Hornbills (F:female, M:male) **(C)**.

After quality control, filtering, and denoising of all samples, a total of 1,868,676 high-quality sequences were obtained, yielding 14,701 OTUs (Supplementary Table 2). OTU-based rarefaction curves indicated that increasing sequencing depth captured more OTUs, and the plateauing curves across all samples demonstrated adequate sequencing coverage for subsequent analyses (Figure 2). All OTU sequences were classified into 44 phyla, 116 classes, 334 orders, 708 families, and 1,855 genera. Specifically, the Oriental Pied Hornbill, Great Hornbill, and Wreathed Hornbill exhibited 5,659, 5,608, and 4,633 OTUs, respectively. Among the three species, 212 OTUs were universally shared, with pairwise comparisons revealing 292 OTUs shared between the Oriental Pied Hornbill and Great Hornbill, 149 between the Oriental Pied Hornbill and Wreathed Hornbill, and 334 between the Great Hornbill and Wreathed Hornbill. Within the Oriental Pied Hornbill group, 204 OTUs were common to both males and females, while 280 OTUs were shared between males and females in the Great Hornbill group (Figure 3).

### 3.2 Analysis of gut microbiota structure

#### 3.2.1 Analysis of gut microbiota composition in three hornbill species

**FIGURE 4:**
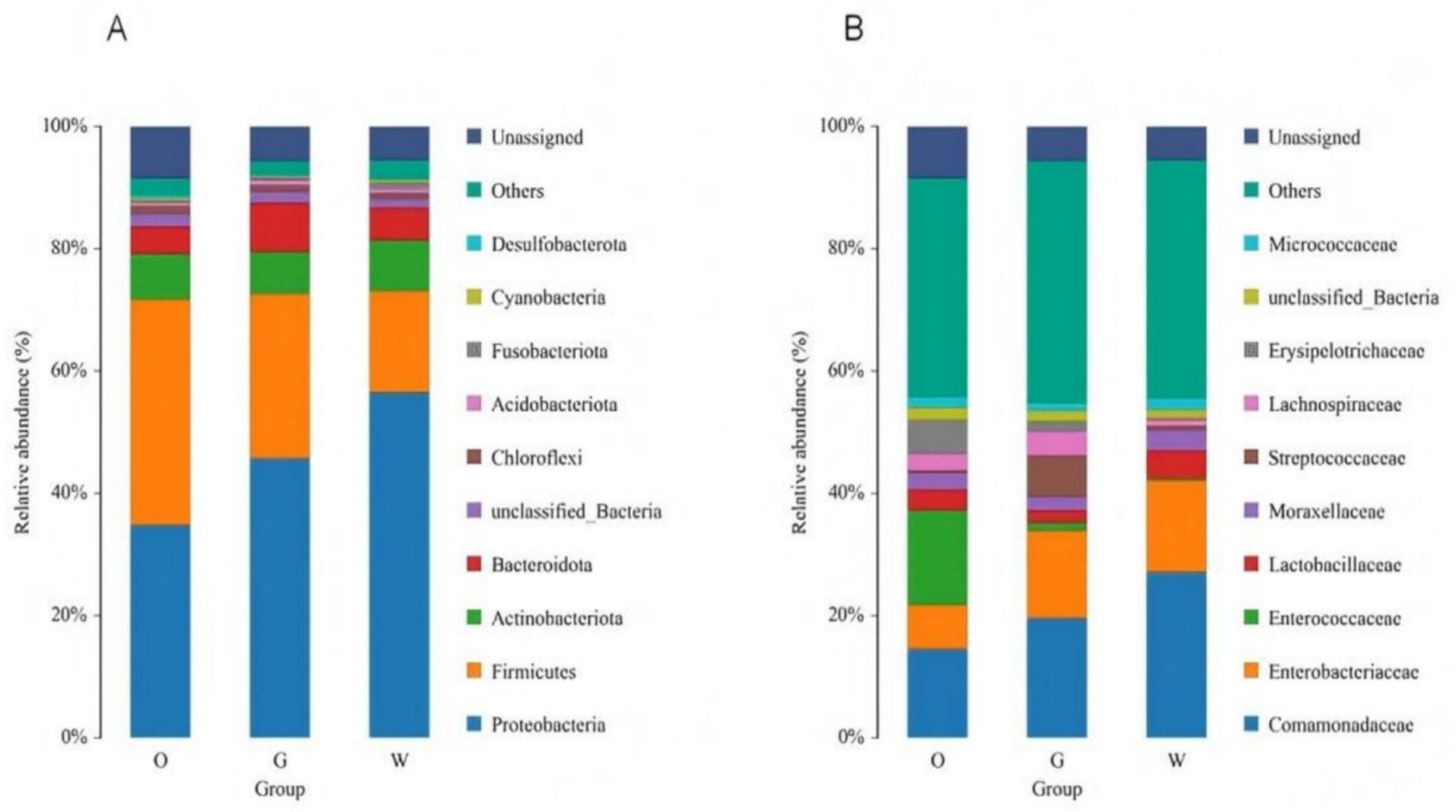
Distribution of the gut microbiota of three hornbill species at the phylum **(A)** and family **(B)** levels.

Based on species annotation results, the top 10 most abundant genera at the phylum level were selected for each sample, and relative abundance bar plots at the phylum level were generated. At the phylum level (Figure 4A), the dominant phyla in the gut of the Oriental Pied Hornbill primarily included Firmicutes (36.99%), Proteobacteria (34.79%), Actinobacteriota (7.40%), and Bacteroidota (4.52%). The gut microbiota of the Great Hornbill was mainly composed of Proteobacteria (45.69%), Firmicutes (27.00%), Bacteroidota (8.05%), and Actinobacteriota (6.85%). The dominant phyla in the gut of the Wreathed Hornbill primarily consisted of Proteobacteria (56.53%), Firmicutes (16.57%), Actinobacteriota (8.32%), and Bacteroidota (5.30%). The abundance of each bacterial phylum varied among the three hornbill species. However, the top four phyla were consistently Proteobacteria, Firmicutes, Actinobacteriota, and Bacteroidota. These four phyla represent the dominant and core phyla within the gut microbiota of the studied hornbill species.

At the family level (Figure 4B), all three hornbill species clearly show a significant proportion of unannotated bacterial taxa. The gut microbiome of the Oriental Pied Hornbill is dominated by Enterococcaceae (15.48%), Comamonadaceae (14.51%), Enterobacteriaceae (7.25%), Erysipelotrichaceae (5.55%), and Lactobacillaceae (3.41%). In the Great Hornbill, the leading families are Comamonadaceae (19.60%), Enterobacteriaceae (14.28%), Lactobacillaceae (6.87%), Micrococcaceae (3.91%), and Lachnospiraceae (2.16%). The Wreathed Hornbill exhibits dominance of Comamonadaceae (27.11%), Enterobacteriaceae (15.01%), Erysipelotrichaceae (4.55%), Lachnospiraceae (3.29%), and Streptococcaceae (1.96%). Notably, variations in the abundance of different bacterial families exist across the hornbill species, with Comamonadaceae emerging as a consistently present and relatively abundant family in all three species.

#### 3.2.2 Analysis of gut microbiota results by sex

**FIGURE 5:**
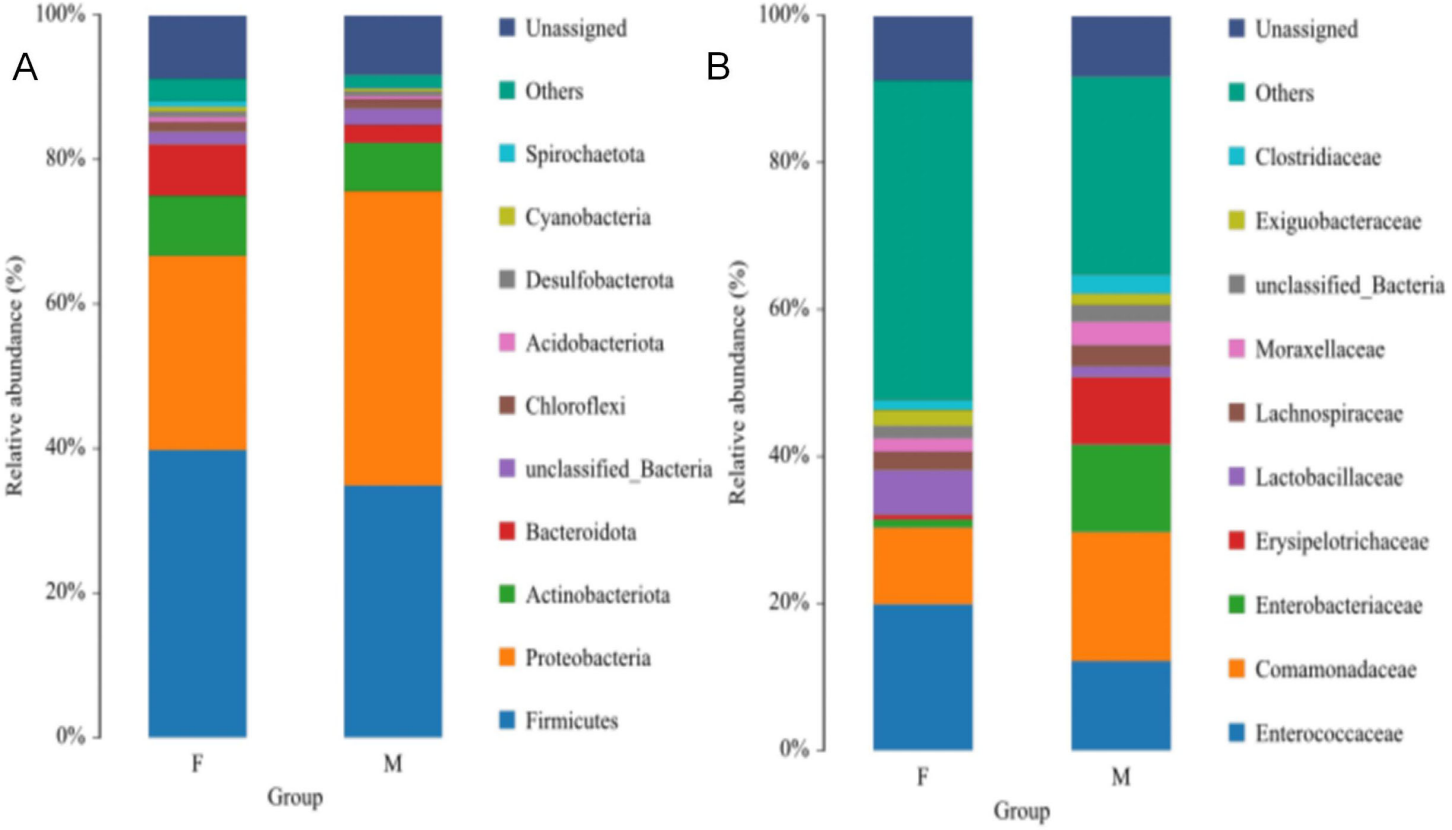
The distribution of intestinal microbiota of Oriental Pied Hornbill at the phylum **(A)** and family **(B)** levels.

**FIGURE 6:**
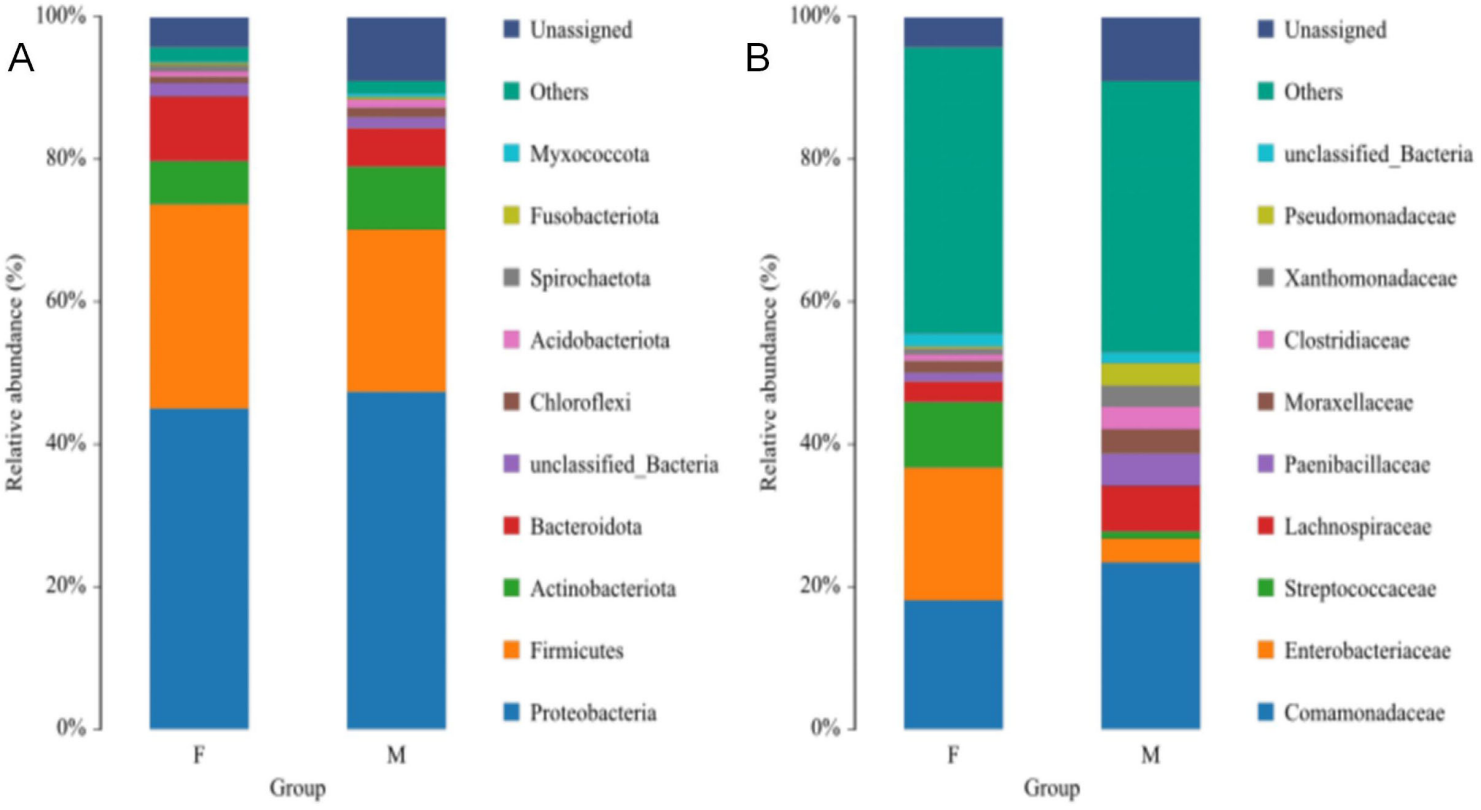
The distribution of intestinal microbiota of Great Hornbill at the phylum **(A)** and family **(B)** levels.

At the phylum level, the dominant phyla in female Oriental Pied Hornbills were Firmicutes (39.78%), Proteobacteria (26.89%), Actinobacteriota (8.34%), and Bacteroidota (7.15%); in males, they were Proteobacteria (40.74%), Firmicutes (34.88%), Actinobacteriota (6.69%), and Bacteroidota (2.53%) (Figure 5A). For female Great Hornbills, the dominant phyla included Proteobacteria (45.01%), Firmicutes (28.69%), Bacteroidota (9.11%), and Actinobacteriota (6.05%); males showed Proteobacteria (47.38%), Firmicutes (22.75%), Actinobacteriota (8.85%), and Bacteroidota (5.39%) as the main phyla. Although the abundance of each bacterial phylum varied across different hornbill species, the top four phyla consistently included Proteobacteria, Firmicutes, Actinobacteriota, and Bacteroidota (Figure 6A).

At the family level, the dominant families in the gut microbiota of female Oriental Pied Hornbills were Enterococcaceae (19.88%), Comamonadaceae (10.46%), Lactobacillaceae (6.03%), Lachnospiraceae (2.48%), and Exiguobacteraceae (2.18%); the dominant families in males were Comamonadaceae (17.56%), Enterococcaceae (12.16%), Enterobacteriaceae (11.91%), Erysipelotrichaceae (9.19%), and Moraxellaceae (3.23%) (Figure 5B). The dominant families in female Great Hornbills were Enterobacteriaceae (18.66%), Comamonadaceae (18.08%), Streptococcaceae (9.19%), Lachnospiraceae (2.91%), and Moraxellaceae (1.67%); in males, the dominant families were Comamonadaceae (23.42%), Lachnospiraceae (6.42%), Paenibacillaceae (4.48%), Moraxellaceae (3.39%), and Enterobacteriaceae (3.30%) (Figure 6B).

### 3.3 Analysis of gut microbiota diversity

#### 3.3.1 Gut microbiota diversity of three hornbill species

Alpha diversity indices, which include species richness estimators and diversity indices (Table 1). The α diversity indices calculated for the Oriental Pied Hornbill, Great Hornbill, and Wreathed Hornbill are as follows: chao1 (486.3097, 630.9528, and 735.1845), ACE (466.8844, 632.2124, and 736.0953), Shannon (5.5506, 5.9427, and 5.4166), and Simpson (0.844, 0.8832, and 0.7619). Pairwise comparisons indicated no significant differences in α diversity among the three hornbill species (*p* > 0.05).

**TABLE 1:** Species richness and diversity indices of intestinal microbiota in three hornbill species.

Species	Chao1	Shannon	Simpson
Oriental Pied Hornbill	486.31 ± 113.55	5.55 ± 0.61	0.84 ± 0.06
Great Hornbill	630.95 ± 97.10	5.94 ± 0.68	0.88 ± 0.05
Wreathed Hornbill	735.18 ± 155.61	5.42 ± 1.03	0.76 ± 0.11

There were no significant differences in richness and diversity indices among the three species groups (*p* > 0.05). Values are presented as least square means ± SEM.

**FIGURE 7:**
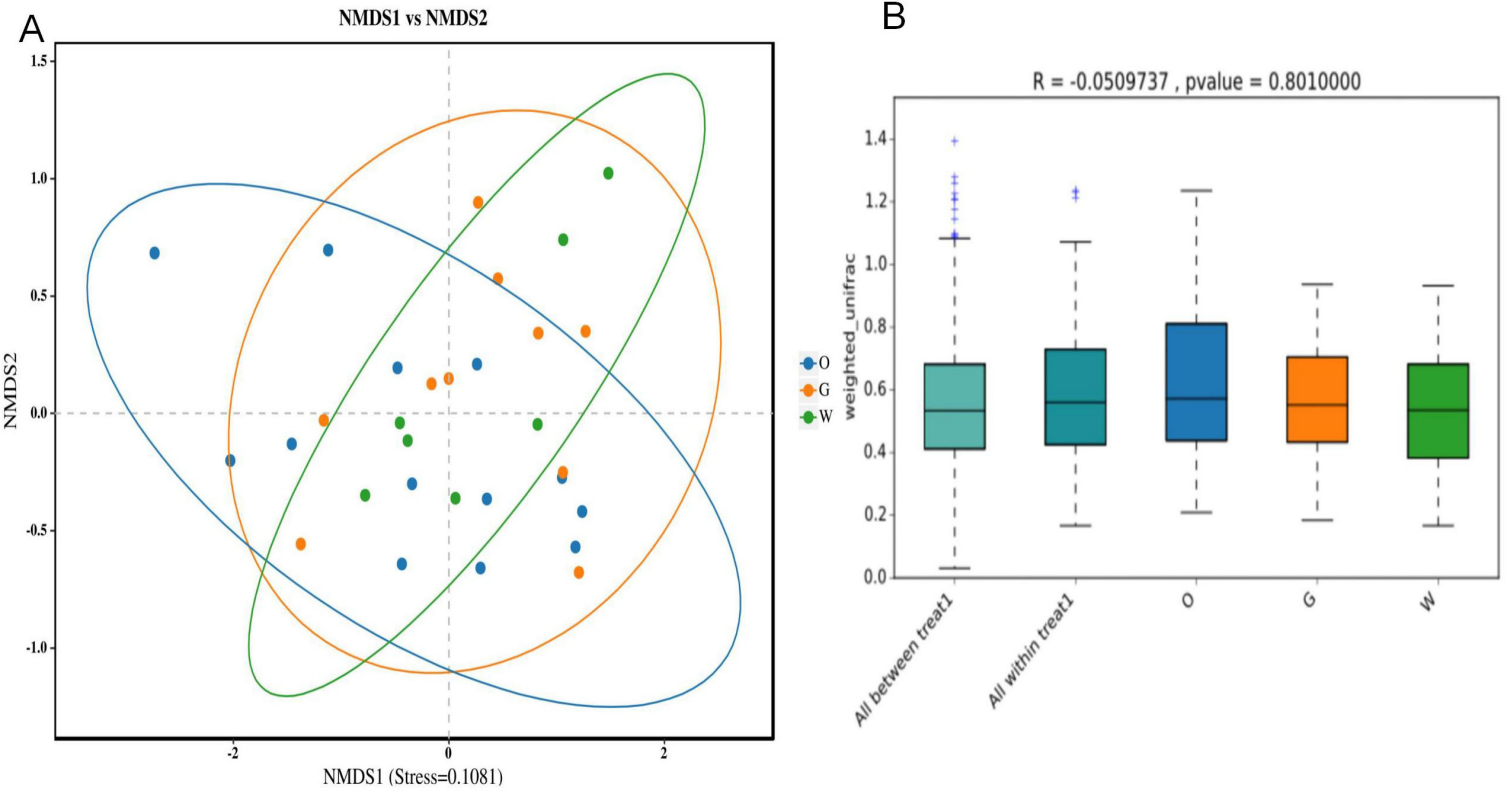
Analysis of differences in intestinal microbiota composition among three hornbill species based on NMDS **(A)** and ANOSIM analyses **(B)**.

Beta diversity analysis revealed no significant separation among the three hornbill samples. The stress value in this analysis was 0.1081 < 0.2, indicating the reliability of the NMDS analysis. Further analysis using ANOSIM yielded an *R* = −0.0509737, *p* = 0.801. This result suggests that intra-group variation exceeded inter-group variation, indicating no significant difference in the gut microbiota composition among the three hornbill species (Figure 7).

#### 3.3.2 Gut microbiota diversity in different sexes

Alpha diversity indices, species richness estimators, and diversity metrics for the gut microbiota of the Oriental Pied Hornbill and Great Hornbill are presented in Table 2. (Note: only two male samples of the Wreathed Hornbill were available, preventing comparative analysis.) The alpha diversity indices for female and male Oriental Pied Hornbills were Chao1 (656.5939, 340.3518), ACE (656.8516, 341.1983), Shannon (6.2331, 4.9657), and Simpson (0.8378, 0.8493), respectively. For female and male Great Hornbills, the alpha diversity indices were Chao1 (606.2926, 688.4933), ACE (607.6718, 689.4739), Shannon (5.6175, 6.7013), and Simpson (0.8532, 0.9532), respectively. Wilcoxon rank sum test indicated no significant differences in alpha diversity between sexes within either the Oriental Pied Hornbill or Great Hornbill (*p* > 0.05).

**TABLE 2:** Species richness and diversity indices of intestinal microbiota in different sexes of Oriental Pied Hornbill and Great Hornbill.

Species	Gender	Chao1	Shannon	Simpson
Oriental Pied Hornbill	F	656.59 ± 196.78	6.23 ± 1.05	0.84 ± 0.12
Oriental Pied Hornbill	M	340.35 ± 111.96	4.97 ± 0.69	0.85 ± 0.06
Great Hornbill	F	606.29 ± 101.37	5.62 ± 0.93	0.85 ± 0.06
Great Hornbill	M	688.49 ± 259.10	6.70 ± 0.75	0.95 ± 0.02

There were no significant differences in richness and diversity indices among the three species groups (*p* > 0.05). Values are presented as least square means ± SEM.

**FIGURE 8:**
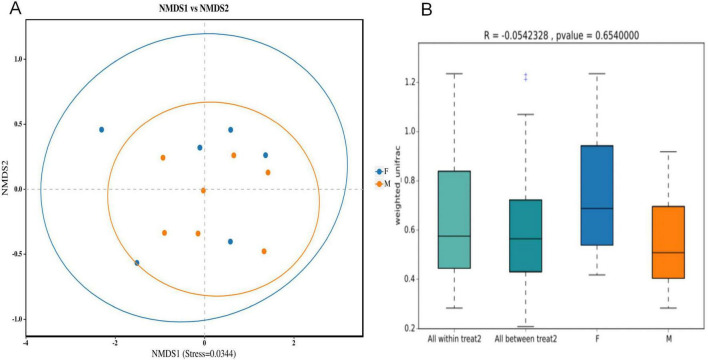
Analysis of differences in intestinal microbiota composition between different sexes of Oriental Pied Hornbill based on NMDS **(A)** and ANOSIM **(B)** analyses.

**FIGURE 9:**
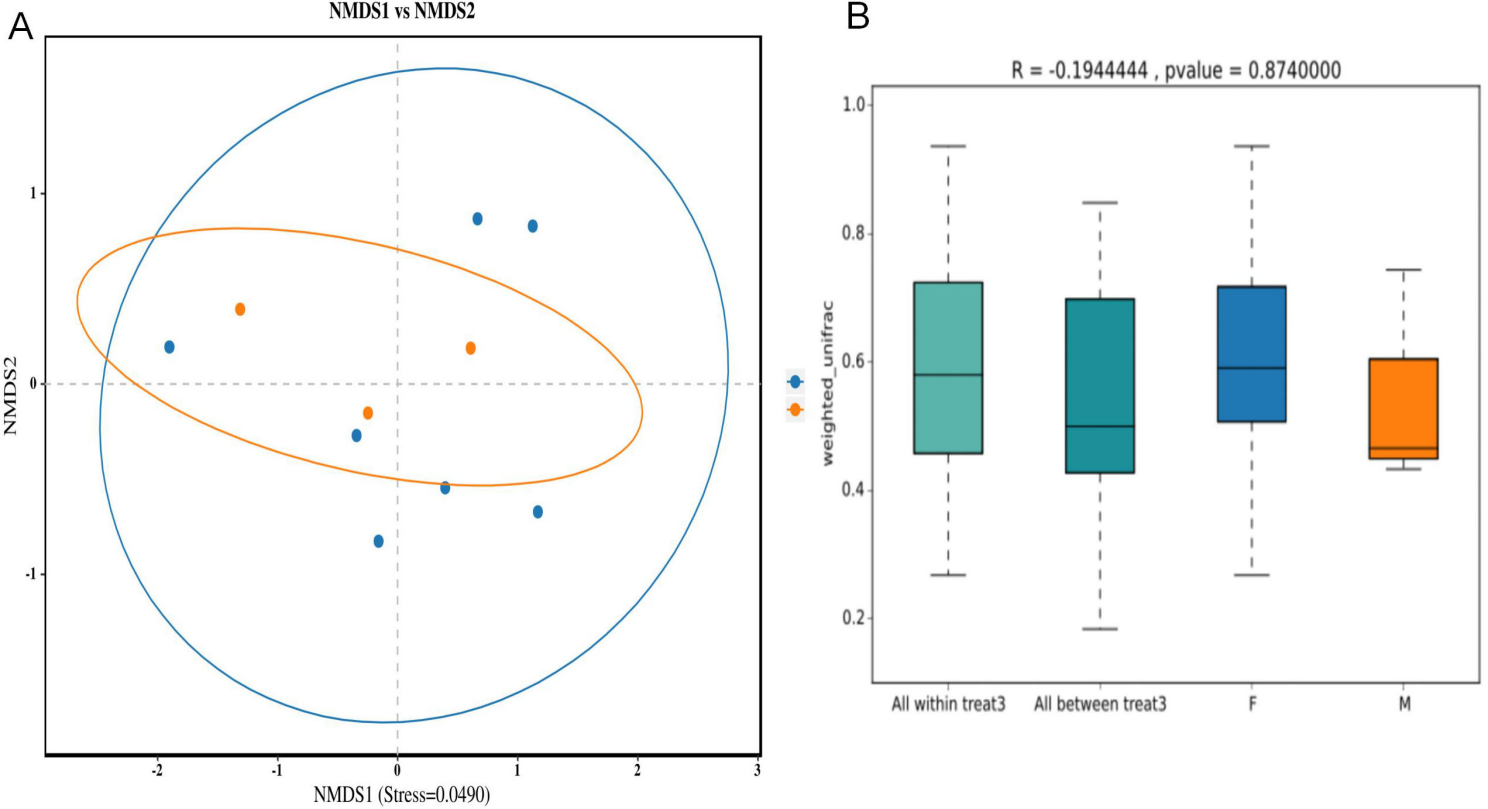
Analysis of differences in intestinal microbiota composition between different sexes of Great Hornbill based on NMDS **(A)** and ANOSIM analyses **(B)**.

NMDS analysis at the OTU level revealed no distinct separation between male and female samples of Oriental Pied Hornbill and Great Hornbill. The stress values for this analysis were 0.0344 and 0.0490, respectively, both less than 0.2, indicating the reliability of the NMDS analysis. Further analysis using ANOSIM yielded *R* = −0.0542328, *P* = 0.654 and *R* = −0.1944444, *P* = 0.874. These results suggest that intraspecific differences between sexes were greater than interspecific differences, indicating no significant difference in gut microbiota composition between males and females of the same hornbill species (Figures 8, 9).

### 3.4 Analysis of intergroup differences in gut microbiota among three hornbill species

**FIGURE 10:**
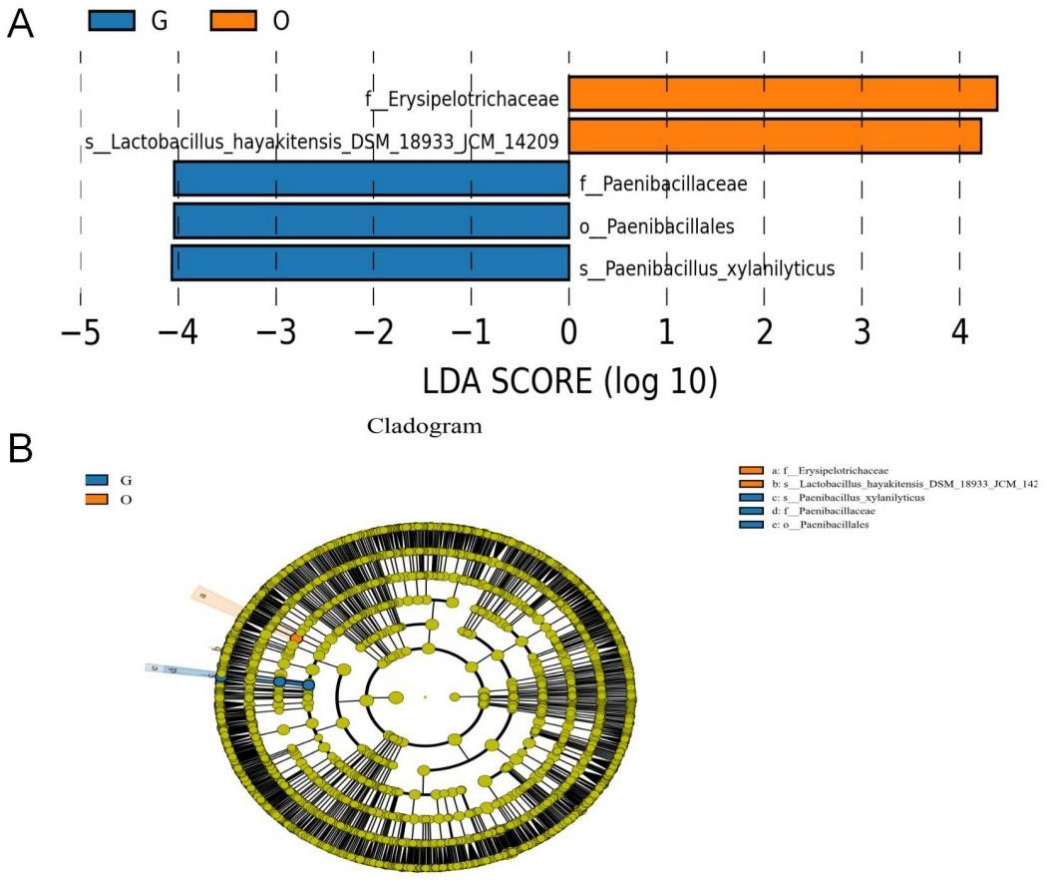
LefSe analysis. Species exhibiting significant differential abundance with LDA scores exceeding the default threshold of 4.0; the length of the histogram bars corresponds to the magnitude of the LDA score, reflecting the impact of taxa with notable differences between groups.**(A)** The cladogram diagram shows the microbial species with significant differences in the three group. Orange and blue indicate different groups, with the species classification at the level of phylum, class, order, family, and genus shown from the inside to the outside. The orange and blue nodes in the phylogenetic tree represent microbial species that play an important role in the O and G groups, respectively. Yellow nodes represent species with no significant difference **(B)**.

LEfSe software was used to analyze the differences in the gut microbiota of three hornbill species, with an LDA score threshold of 4. The longer the histogram, the greater the impact of species differences. The evolutionary branch icon annotates the biomarkers with significant differences in gut microbiota between groups (Figure 10). Erysipelotrichaceae at the family level and Lactobacillus hayakitensis DSM_18933_JCM_14209 at the species level showed significant differences and were enriched in the Oriental Pied Hornbill group. Paenibacillales at the order level, Paenibacillaceae at the family level, and Paenibacillus xylanilyticus at the species level demonstrated significant differences and were enriched in the Great Hornbill group. No significantly different species were annotated in the Wreathed Hornbill group.

### 3.5 Functional prediction of gut microbiota in three hornbill species

**FIGURE 11:**
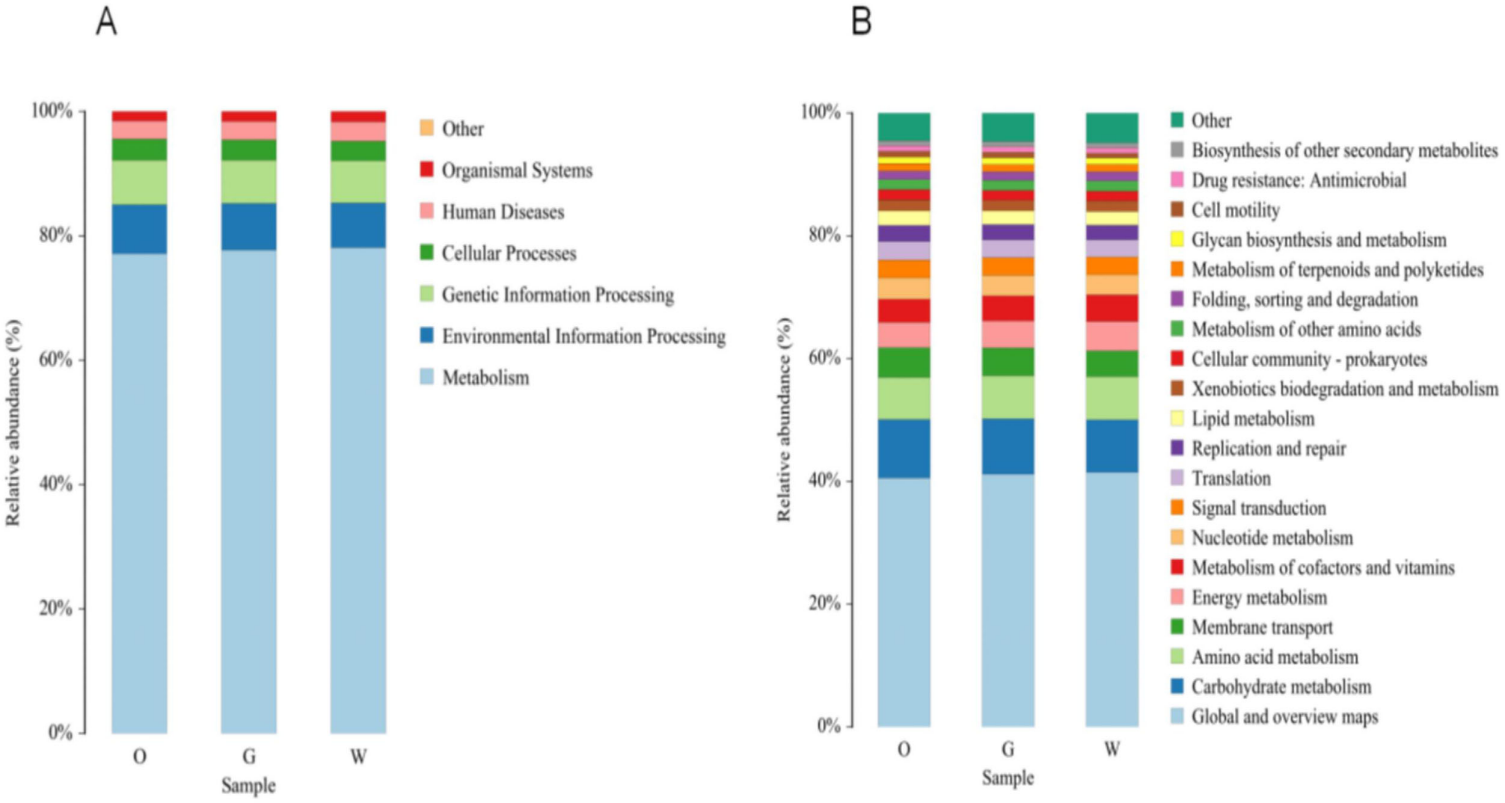
Proportion of primary pathway functions **(A)** and secondary pathway functions **(B)** in gut microbiota samples of different hornbill species.

PICRUSt analysis was used to investigate the functional potential of gut microbial communities in three hornbill species. Predicted functional genes were accurately mapped to six level-one pathways and 39 level-two pathways within the KEGG database (Figure 11). The level-one pathways revealed six core functional domains: metabolism, environmental information processing, genetic information processing, cellular processes, human diseases, and organismal systems. Notably, approximately 80% of the functional genes were enriched in the metabolism pathway. Further analysis at the level-two pathway indicated that genes were primarily enriched in metabolism-related pathways, with carbohydrate metabolism, amino acid metabolism, and energy metabolism being dominant. Additionally, genes related to genetic information processing (e.g., replication and repair, translation) and environmental information processing (e.g., membrane transport, signal transduction) were also abundant. Analysis of the top 20 level-two pathways indicated similar relative abundances of functional genes among the three hornbill species.

## 4 Discussion

This study analyzed the gut microbiota composition and diversity of three hornbill species under artificial captive environment. We found no significant differences in the gut microbiota structure among the three hornbill species and identified their dominant taxonomic units, while also predicting the functions of the gut microbial communities of the three hornbill species.

The dominant phyla in the gut microbiota of three hornbill species were Proteobacteria, Firmicutes, Actinobacteria, and Bacteroidetes. This finding is consistent with previous research on the gut microbiota of omnivorous birds (Grond et al., 2014). These phyla are also prevalent in the gut microbiota of mammals, such as pigs (Lowe et al., 2012) and horses (O’Donnell et al., 2013). Furthermore, Firmicutes and Proteobacteria have also been identified as the most abundant bacterial phyla in the guts of other scavenging birds, such as vultures (Roggenbuck et al., 2015), herbivorous birds, such as geese (Wang et al., 2018) and carnivorous birds, such as penguins (Dewar et al., 2014), which may be highly related to the function of these phyla. The Proteobacteria phylum was identified as the predominant intestinal microbial group in both the Great Hornbill and the Wreathed Hornbill in this study, serving as the dominant bacterial phylum in these two hornbill species at Hongshan Zoo. This phylum encompasses a diverse array of bacterial species, among which beneficial bacteria demonstrate metabolic flexibility to prevent intestinal diseases and play crucial roles in certain physiological and biochemical functions within avian intestines. These significant microorganisms may potentially be passed down through generations. However, an increase in certain pathogenic bacteria is considered a marker of dysbiosis and disease risk (Shin et al., 2015). The dominant phylum in the intestinal flora of the Great Hornbill is Firmicutes, which promotes the absorption of fatty acids and aids in the degradation of dietary fiber, converting it into volatile fatty acids for host utilization. This strengthens the link between the host’s gut microbiota and its energy nutritional status (Guo et al., 2024). The abundance of Firmicutes in the Oriental Pied Hornbill gut microbiota is higher than that in the Great Hornbill and the Wreathed Hornbill, which may be attributed to differences in host dietary habits and physiological characteristics. It is speculated that the Great Hornbill prefers high-fat foods; therefore, lipids and fiber-rich foods should be increased during artificial feeding. Additionally, the phyla Actinobacteria and Bacteroidetes were present in the gut of all three species. Research has shown that Bacteroidetes can have a probiotic effect on animals by promoting the degradation and absorption of polysaccharides and proteins, accelerating nutrient utilization, and maintaining intestinal micro-equilibrium (Bäckhed et al., 2004). Furthermore, Bacteroidetes can degrade organic substances such as proteins, lipids, and polysaccharides and participate in their carbon conversion, making them a key factor in the mineralization of organic carbon and the carbon cycle in aquatic environments (Schwalm and Groisman, 2017). Actinobacteria are often found in environments such as soil and water, and the abundance of Actinobacteria in the animal gut may be related to the host’s intake of cellulose (Lee et al., 2015). It is therefore speculated that the presence of these two phyla can help hornbills increase the degradation and absorption of polysaccharides, proteins, and cellulose. Previous studies on the gut microbiota of the Great Hornbill and the Wreathed Hornbill revealed the presence of Cyanobacteria and Fusobacteria (Sun et al., 2019), which were not detected in this study. The current research was conducted at the Nanning Zoo, where the three hornbill species—Great Hornbill, Wreathed Hornbill, and Oriental Pied Hornbill—were primarily fed rice balls (rice, minced pork, cooked separately), plantains, tomatoes, grapes, apples, and mealworms. In contrast, Sun et al.’s research was conducted at the Nanjing Hongshan Zoo, where the primary diet consisted of rice balls (rice, beef, eel, eggs, and carrots, cooked separately), bananas, cherries, tomatoes, grapes, and watermelon. The fruits and vegetables provided as food in our experiment differed from those in the previous study. Furthermore, previous research has indicated that variations in the relative abundance of dominant bacterial phyla among birds may be influenced by dietary factors (Xie et al., 2016). Therefore, we hypothesize that the primary reason for the discrepancies between our study and the findings of Sun et al. may be attributed to differences in dietary composition.

This study performed alpha and beta diversity analyses of the gut microbiota of three different hornbill species and sexes. The results indicated that there were no significant differences in the gut microbiota composition among different species and sexes. Similar phenomena have been observed in studies of other species. Loo analyzed nine finch species occupying different ecological niches, using Darwin’s finches as a model. They found that the composition of their gut microbiota was primarily driven by environmental filtering (through diet and habitat), while the direct influence of host evolutionary history was relatively weak (Loo et al., 2019). Furthermore, a study of two high-altitude bird species, the Kentish plover (*Charadrius alexandrinus*) and the Tibetan sand plover (*Charadrius altrifrons*), found no significant differences in gut microbiota when considering only species, age, and sex. However, the gut microbiota was affected by the interaction between age and sex (Sun et al., 2024). Since the age information of the experimental individuals in this study could not be determined, the impact of age on the gut microbiota requires further investigation.

It is noteworthy that a recent study on the role of gut microbiota in how animals adapt to extreme environments indicates that the Yunnan snub-nosed monkey (Rhinopithecus bieti), which lives in cold, high-altitude regions, and the reindeer (Rangifer tarandus valentinae), which lives in the Arctic region, two distantly related species, have evolved similar adaptive mechanisms in their gut microbiota (Yao et al., 2025). This further illustrates that the gut microbiota of different species in similar environments may exhibit certain similarities. Food resources in similar environments are also usually similar, as the results of this study further validate the view that the composition of gut microbiota is closely related to food resources. In this study, three hornbills were kept in similar cages, with the same artificial diet, and the same feeding environment and artificial operations. These factors may have weakened the influence of host evolution or physiological differences on the microbiota while becoming the main reason for the lack of significant differences in the gut microbiota of the three hornbills.

## Data Availability

The data presented in the study are deposited in the NCBI SRA repository; accession number PRJNA1297384.

## Footnotes

1
https://www.forestry.gov.cn/c/www/lczc/90131.jhtml
