# Correction: Comparative analysis of the gut microbiota composition among three captive hornbills

**DOI:** 10.3389/fmicb.2026.1806153

**Published:** 2026-03-26

**Authors:** Enmei Yang, Song Wang, Huajuan Feng, Fanglin Zheng, Yubao Duan, Shuang Yang

**Affiliations:** 1 The Key Laboratory of Forest Resources Conservation and Utilization in the Southwest Mountains of China Ministry of Education, Southwest Forestry University, Kunming, China; 2 Key Laboratory of National Forestry and Grassland Administration on Biodiversity Conservation in Southwest China, Southwest Forestry University, Kunming, China; 3 Key Laboratory for Forest Resources Conservation and Utilization in the Southwest Mountains of China, Ministry of Education, Southwest Forestry University, Kunming, China; 4 Key Laboratory of Forest Disaster Warning and Control in Yunnan Province, Southwest Forestry University, Kunming, China; 5 Key Laboratory for Conserving Wildlife with Small Populations in Yunnan, College of Forestry, Southwest Forestry University, Kunming, China; 6 Nanning Zoo, Nanning, China

**Keywords:** hornbills, gut microbiota, diversity, 16s rRNA sequencing technology, gut microbiota composition

In the published article, the description of the number of OTUs shared among the three species contained an error.

A correction has been made to the section 3 **Results and analysis**, subsection 3.1 *Sequencing results statistics*, Paragraph 3:

“After quality control, filtering, and denoising of all samples, a total of 1,868,676 high-quality sequences were obtained, yielding 14,701 OTUs (Supplementary Table 2). OTU-based rarefaction curves indicated that increasing sequencing depth captured more OTUs, and the plateauing curves across all samples demonstrated adequate sequencing coverage for subsequent analyses (Figure 2). All OTU sequences were classified into 44 phyla, 116 classes, 334 orders, 708 families, and 1,855 genera. Specifically, the Oriental Pied Hornbill, Great Hornbill, and Wreathed Hornbill exhibited 5,659, 5,608, and 4,633 OTUs, respectively. Among the three species, 212 OTUs were universally shared, with pairwise comparisons revealing 504 OTUs shared between the Oriental Pied Hornbill and Great Hornbill, 361 between the Oriental Pied Hornbill and Wreathed Hornbill, and 546 between the Great Hornbill and Wreathed Hornbill. Within the Oriental Pied Hornbill group, 204 OTUs were common to both males and females, while 280 OTUs were shared between males and females in the Great Hornbill group (Figure 3).”

